# The AOP-DB RDF: Applying FAIR Principles to the Semantic Integration of AOP Data Using the Research Description Framework

**DOI:** 10.3389/ftox.2022.803983

**Published:** 2022-02-14

**Authors:** Holly M. Mortensen, Marvin Martens, Jonathan Senn, Trevor Levey, Chris T. Evelo, Egon L. Willighagen, Thomas Exner

**Affiliations:** ^1^ United States Environmental Protection Agency, Office of Research and Development, Center for Public Health and Environmental Assessment, Research Triangle Park, Durham, NC, United States; ^2^ Department of Bioinformatics (BiGCaT), Maastricht University, Maastricht, Netherlands; ^3^ Oak Ridge Associated Universities, Oak Ridge, TN, United States; ^4^ SAS Institute, Cary, NC, United States; ^5^ Maastricht Centre for Systems Biology, Maastricht University, Maastricht, Netherlands; ^6^ Seven Past Nine, Cerknica, Slovenia

**Keywords:** semantic web, adverse outcome pathway, toxcast assays, disease, pathway, ontological mapping

## Abstract

Computational toxicology is central to the current transformation occurring in toxicology and chemical risk assessment. There is a need for more efficient use of existing data to characterize human toxicological response data for environmental chemicals in the US and Europe. The Adverse Outcome Pathway (AOP) framework helps to organize existing mechanistic information and contributes to what is currently being described as New Approach Methodologies (NAMs). AOP knowledge and data are currently submitted directly by users and stored in the AOP-Wiki (https://aopwiki.org/). Automatic and systematic parsing of AOP-Wiki data is challenging, so we have created the EPA Adverse Outcome Pathway Database. The AOP-DB, developed by the US EPA to assist in the biological and mechanistic characterization of AOP data, provides a broad, systems-level overview of the biological context of AOPs. Here we describe the recent semantic mapping efforts for the AOP-DB, and how this process facilitates the integration of AOP-DB data with other toxicologically relevant datasets through a use case example.

## Introduction

There is a need for more efficient use of existing data through improved data integration and compatibility of data structures to characterize human toxicological response data for environmental chemicals. Assessors in the US are moving towards the use of existing mechanistic data (*in vitro* and *in silico*) that provide insights into adverse outcomes in humans (National Research Council (NRC), 2007; National Research Council (NRC), 2009; National Research Council (NRC), 2010; (National Research Council (NRC), 2017), and reduced animal testing (Wheeler, 2019). The Adverse Outcome Pathway (AOP) framework helps to organize existing mechanistic information and contributes to what is currently being described as New Approach Methodologies (NAMs) (Thomas et al., 2019). The US EPA Adverse Outcome Pathway-Database (AOP-DB) is a decision support tool for risk assessors, developed by the EPA’s Center for Public Health and Environmental Assessment, which contributes to NAMs (e.g., computational toxicology tools) used for the Toxic Substances Control Act (Public Law 114–182, 2016). The AOP-DB has been made available through the Office of Science Management as a public EPA database since November 2021. Pertinent AOP-DB data is currently integrated with the CompTox Chemicals Dashboard (https://comptox.epa.gov/dashboard/chemical_lists/AOPSTRESSORS), which maps the Distributed Structure-Searchable Toxicity records to the most current list of AOP-DB stressors.

**FIGURE 1:**
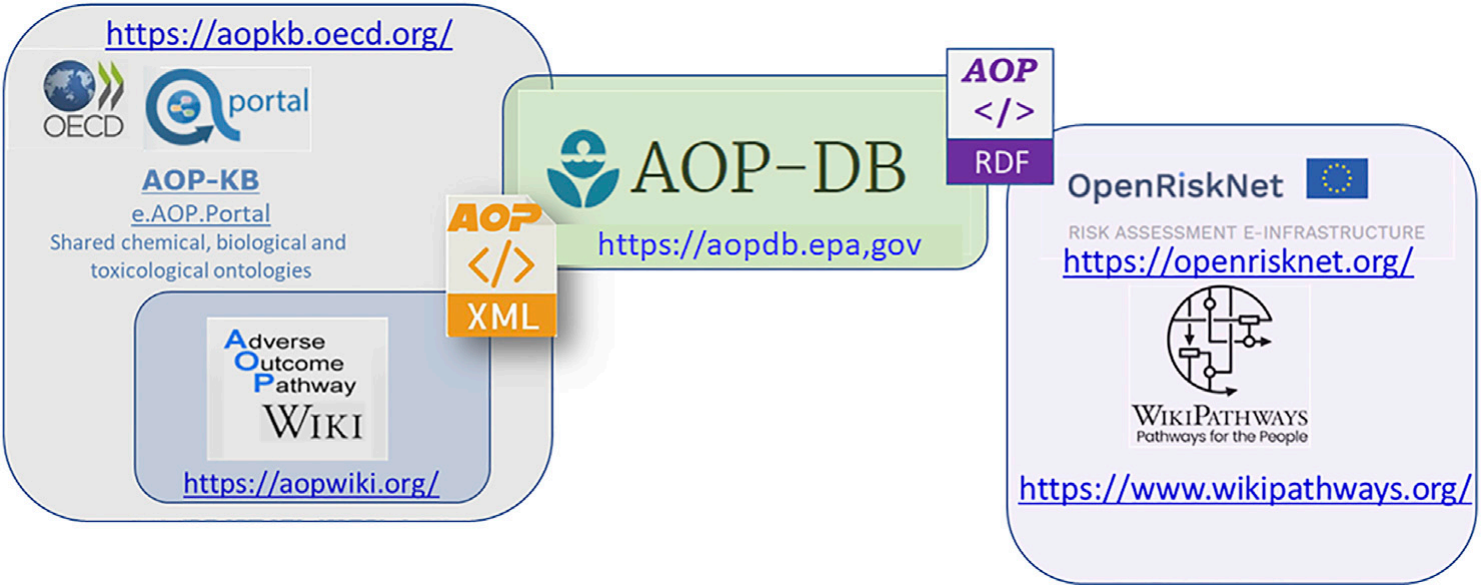
The OECD funded AOP-KB currently support the AOP-Wiki. The EPA AOP-DB, currently slated as a third-party tool for integration with the AOPKB 2.0, automatically and programmatically pulls AOP data from the AOP-KB XML, and extends AOP capabilities to users with semantic resources like WikiPathways and the OpenRiskNet e-infrastructure that incorporate the Research Description Framework (RDF). Integration of data across the AOP-KB (AOP-Wiki), AOP-DB, and expanding research frameworks through WikiPathways and the EU funded OpenRiskNet, creates additional ontological linkages and improves capabilities for computational analyses. These tools are useful to AOP users trying to retrieve information for AOP development, as well as those trying to understand and characterize existing AOPs.

The AOP-DB integrates AOP content to help users characterize AOPs from the OECD-funded AOP-KB (https://aopkb.oecd.org/index.html) effort, where the AOP-Wiki (https://aopwiki.org/) is the primary repository for direct user submission of AOP information to the AOP-KB. Because the AOP-Wiki data is challenging to parse in its current format (Ives et al., 2017; Martens et al., 2018), the AOP-DB was developed to assist in automating and organizing AOP data, as well as integrating with publicly available datasets to allow biological and mechanistic characterization of AOPs and provide a systems-level overview of the biological context of AOPs (Mortensen et al., 2018; Pittman et al., 2018). Recent updates to AOP-DB in version 2 (Mortensen, 2021; Mortensen et al., 2021) include 280 AOPs (1,111 kEs) from the AOP-Wiki XML. The semantic mapping of AOP-DB data, described herein, extends AOP capabilities to users through the incorporation of the Research Description Framework (RDF), which creates additional ontological linkages and improves capabilities for computational analyses (Figure 1). These tools are useful to AOP users trying to retrieve information for AOP development or to understand and characterize existing AOPs. Here we describe the recent semantic mapping efforts for the AOP-DB, and how this process integrates AOP-DB data with other toxicologically relevant datasets.

As part of OpenRiskNet, a 3 years project supported by the European Commission within Horizon2020 EINFRA-22-2016 Programme, the US EPA AOP-DB was selected as an Implementation Challenge winner. The Implementation Challenge was created to select external tools for use in risk assessment to be prioritized for integration in the OpenRiskNet e-Infrastructure (https://openrisknet.org/) and foster collaborative interaction between project partners. In contribution to this effort, US EPA and Maastricht University project partners have completed the semantic mapping of several AOP-DB data tables into RDF, which is a standard model for data interchange (W3C, 2014). The application of RDF defines relationships between data objects using triplestores that include three positional statements (subject, predicate and object). The mapping of AOP-DB data to the RDF data model stores relevant AOP information in a computer-readable format, and contributes to the identification, disambiguation, and meaningful linkage of AOP data with other data structures, following FAIR (findable, accessible, interoperable, and reusable) principles (Wilkinson et al., 2019a; Wilkinson et al., 2019b).

## Materials and Methods

We selected seven AOP-DB data tables for semantic integration, specifically the Gene Interaction, Biological Pathway, Toxcast Assay, Taxonomy, Chemical-Gene, Gene Info, and Key Event tables. In developing the AOP-DB RDF, we implemented the most recent version of the SQL AOP-DB (Mortensen, 2020) to map each table of interest into RDF triples. Each table was filtered using the R version 3.6 and Rstudio version 1.2.83 (R Core Team, 2020) to include only records involving a molecular initiating event (MIE) or key event (KE) that maps to a molecular identifier (e.g., gene, protein, cytokine). Code was developed to implement each record as input, modify and filter the AOP-DB table data, and output each modified record to an RDF triple. Additionally, subjects were created for Ensembl and UniProt identifiers. Ontology terms were referenced using BioPortal (Whetzel et al., 2011) in order to find the most appropriate ontology terms for each entity, in line with the AOP-Wiki RDF (Martens et al., 2021a) for optimal interoperability between the two resources. Terms were selected with the most accurate description from ontologies that are relevant to the context of the field. For the development of the AOP-DB RDF, several ontologies and consistent vocabularies have been included. Furthermore, publicly available datasets included in the AOP-DB for RDF mapping are described in detail in Mortensen *et al.* (2021). Table 1 provides an overview of the included ontologies and database links, including their prefix in the RDF and their corresponding Internationalized Resource Identifier (IRI).

**TABLE 1:** Overview of ontologies, consistent vocabularies and databases included in the AOP-DB RDF.

Ontologies and Vocabularies		
Name	Prefix in RDF	IRI
AOP Ontology Burgoon, (2017)	Aopo	http://aopkb.org/aop_ontology#
BioAssay Ontology Abeyruwan et al. (2014)	Bao	http://www.bioassayontology.org/bao#
Chemical Information ontology Hastings et al. (2011)	Cheminf	http://semanticscience.org/resource/CHEMINF_
Dublin Core	dc	http://purl.org/dc/elements/1.1
EDAM Ontology Ison et al. (2013)	edam	http://edamontology.org
Friend Of A Friend	foaf	http://xmlns.com/foaf/0.1
Logical Observation Identifier Names and Codes McDonald et al. (2003)	loinc	http://purl.bioontology.org/ontology/LNC
Molecular Interactions Millán, (2020)	mi	http://purl.obolibrary.org/obo/MI_
Measurement Method Ontology Smith et al. (2013)	mmo	http://purl.obolibrary.org/obo/MMO_
NCBI Taxonomy Bodenreider, (2004)	ncbitaxon	http://purl.bioontology.org/ontology/NCBITAXON
Pathway Ontology Petri et al. (2014)	pw	http://purl.obolibrary.org/obo/PW_
RDF Schema	rdfs	http://www.w3.org/2000/01/rdf-schema#
Semantics Science Ontology Dumontier et al. (2014)	sio	http://semanticscience.org/resource
Simple Knowledge Organization System	skos	http://www.w3.org/2004/02/skos/core#
Uber Anatomy Ontology Mungall et al. (2012)	uberon	http://purl.obolibrary.org/obo/UBERON_
Databases		
AOP-Wiki	aop.events	http://identifiers.org/aop.events
Comptox Dashboard Williams et al. (2017)	assay	https://comptox.epa.gov/dashboard/assay_endpoints
CAS Common Chemistry	cas	https://identifiers.org/cas
Ensembl Yates et al. (2020)	ensembl	http://identifiers.org/ensembl
HUGO Genome Nomenclature Committee Braschi et al. (2019)	hgnc	https://identifiers.org/hgnc
NCBI Gene	ncbigene	https://identifiers.org/ncbigene
Uniprot UniProt Consortium (2019)	uniprot	https://identifiers.org/uniprot
KEGG Pathways Kanehisa et al. (2021)	kegg.pathway	https://identifiers.org/kegg.pathway
PharmGKB Pathways Whirl-Carrillo et al. (2021)	pharmgkb.pathways	https://identifiers.org/pharmgkb.pathways
Small Molecule Pathway Database Jewison et al. (2014)	smpdb	https://identifiers.org/smpdb
BioCyc Karp et al. (2019)	biocyc	https://identifiers.org/biocyc
BioCarta Pathways	biocarta.pathway	https://identifiers.org/biocarta.pathway
Reactome Jassal et al. (2020)	reactome	https://identifiers.org/reactome
NCI Pathway Interaction Database Schaefer et al. (2009)	pid.pathway	https://identifiers.org/pid.pathway
NetPath Kandasamy et al. (2010)	netpath	http://netpath.org/pathways?path_id=
WikiPathways Martens et al. (2021b)	wikipathways	https://identifiers.org/wikipathways
AOP-DB Chemical-Gene association	chemicalgeneassociation	http://example.org/ChemicalGeneAssociation
AOP-DB Protein Interaction	proteinInteraction	http://example/proteinInteraction

### Testing the AOP-DB RDF

Using a Jupyter notebook (Jupyterlab version 3.2.5, Python version 3.8.5), the AOP-DB SPARQL endpoint has been tested by executing SPARQL queries, using the SPARQLWrapper Python library (version 1.8.5). SPARQL queries were used to extract statistics of the data, and a federated SPARQL query was constructed to explore the integrative capabilities of the AOP-DB RDF. The Jupyter notebook, SPARQL queries for extracting data counts, and instructions for setting up the AOP-DB SPARQL endpoint are available on https://github.com/BiGCAT-UM/AOP-DB-RDF.

## Results

### The AOP-DB Semantic Mapping

**FIGURE 2:**
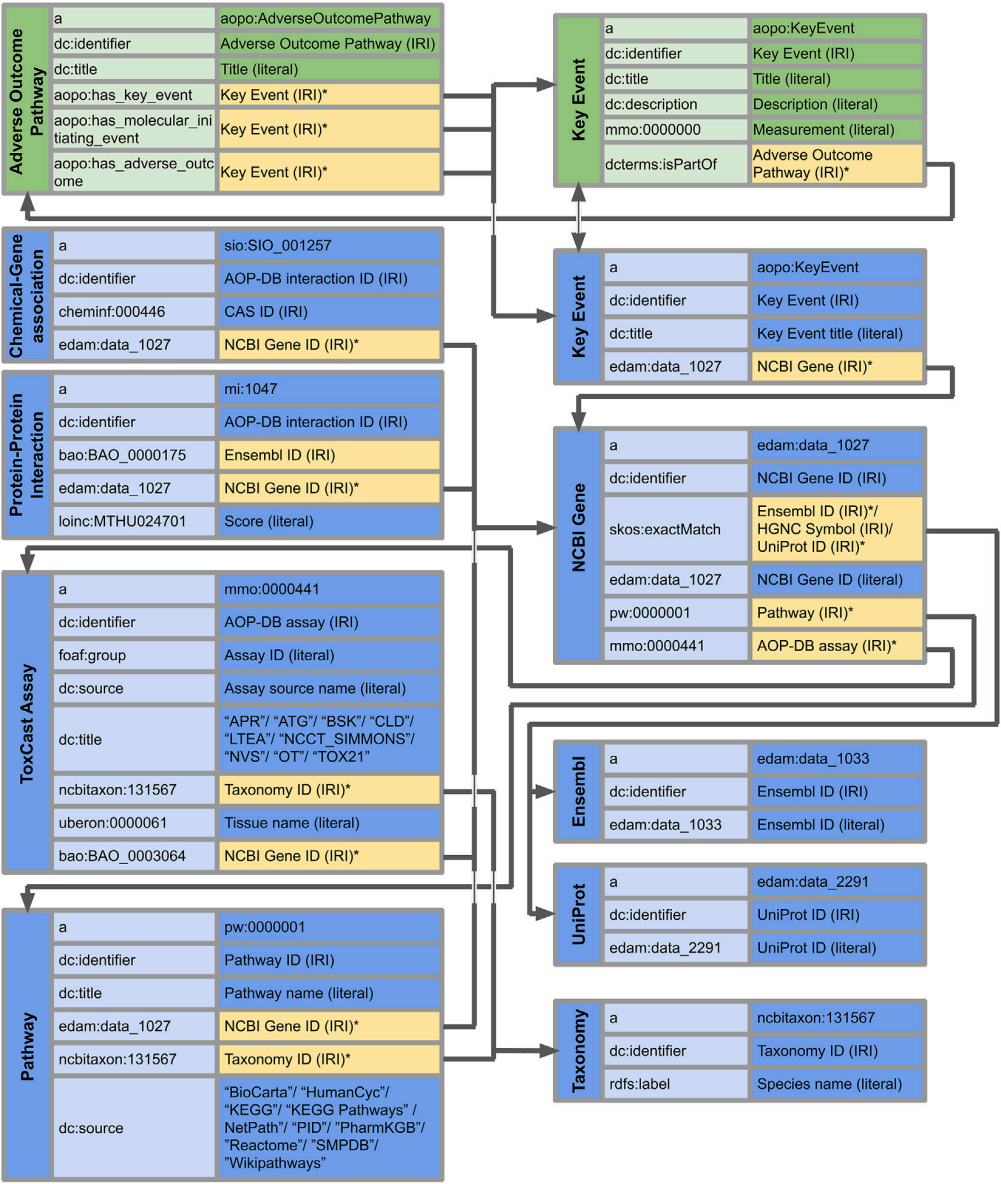
AOP-DB Semantic Mapping using illustrating the predicates and objects of the nine core subject types in the AOP-DB RDF (in blue). Vertical columns show subjects, and the middle and right columns indicate predicates and objects, respectively. Where applicable, the type of entry is indicated (literal or IRI). Yellow objects with an asterisk (*) indicate the connection between their subjects and the subjects of other tables. The interaction with the AOP-Wiki RDF is highlighted at the Key Events and Adverse Outcome Pathways (in green). Forward slashes indicate the inclusion of multiple objects as part of the subject-predicate-object triple.

The AOP-DB RDF schema developed according to the methods described above resulted in the primary and secondary table structure, as illustrated in Figure 2. The AOP-DB extends AOP-Wiki RDF with the inclusion of gene/protein, chemical, ToxCast, and biological pathway and taxonomy information. In total, the RDF contains 157 kEs, 376 NCBI genes linked to KEs, 93,449 Chemical-Gene Interactions (3,982 unique chemicals and 122 unique genes), 763,446 Protein-Protein Interactions, 1,143 ToxCast Assays 110,833 Biological Pathways from 10 sources, and 22 taxonomies. Also, the NCBI Gene IDs were matched to 299 Ensembl IDs and 1,026 UniProt IDs. The AOP-DB RDF data tables associate the gene and protein information of AOP genes to chemical, pathway, and assay information organized within the AOP-DB (Mortensen, 2020; Mortensen, 2021).

The Key Event subjects are linked to NCBI Genes through the ‘data_1,027’ term of the EDAM ontology, which in turn is linked to pathways and assays with respectively the terms ‘pw:0000001’ from the Pathway Ontology and ‘mmo:0000441’ from the Measurement Method Ontology. Furthermore, matching identifiers were linked with ‘skos:exactMatch’, providing IRIs of Ensembl IDs, HGNC Symbols, and UniProt IDs. On the other hand, Chemical-Gene interactions, Protein-protein interactions, ToxCast assays, and Pathways have links to NCBI Gene subjects through the term ‘data_1,027’ from the EDAM ontology. Finally, taxonomy is referenced by ToxCast assay and pathway subjects through the term ‘ncbitaxon:131,567’ indicating cellular organism.

### The AOP-DB SPARQL Endpoint

The AOP-DB RDF can be explored through the AOP-DB SPARQL (https://aopdb.rdf.bigcat-bioinformatics.org/sparql). It allows custom SPARQL queries to return output tables in a variety of formats, where it is possible to directly combine different resources with federated SPARQL queries.

### AOP-DB RDF Use Case Example

SPARQL queries can be used to query the RDF in order to answer biological and toxicological questions, such as which molecular targets (e.g. genes/proteins), chemical stressors, key events, or *in vitro* assays are relevant for adverse outcomes of interest. The use case examples provided herein (Supplementary 1) illustrate the utility of the AOP-DB RDF content, as well as the power of integrating these data with other diverse, external databases using federated queries. Our first use case implements the AOP-DB RDF to identify AOP-relevant molecular targets that have associated ToxCast assay targets, which has previously not been possible. The automated linkage of ToxCast assays and KEs in AOP-Wiki can serve as a prioritization tool by exploring the activation of KEs by the many chemicals that have been investigated in ToxCast. The second use case shows the integration of the AOP-DB RDF with other databases that provide access to their data through SPARQL endpoints. A single SPARQL query can be executed to extract AOP IDs, KE IDs, KE titles and protein names from the AOP-Wiki RDF, extract protein descriptions from the Protein Ontology, and the names and descriptions of pathways in WikiPathways, all based on the NCBI Gene IDs captured in the AOP-DB. Through the integration of these diverse data sources, we can effectively explore the data and build automated computational workflows to address questions of toxicological concern.

## Conclusion

A central goal of computational toxicology is to predict and explain how the human body responds after exposure to specific xenobiotics or other chemicals *in silico.* This effort has been hampered by several major limiting factors, including fragmented and poorly structured data, and insufficient access to computational resources and expertise. The AOP-DB RDF and SPARQL endpoint created and discussed herein allow improved access to rigorously structured AOP data and other associated data of toxicological interest. This work improves computational organization and efficiency, through improved data integration, for toxicological and related datasets, and contributes to continued progress in computational toxicology, chemical screening and the improvement of human health risk assessment.

The AOP-DB RDF will be improved with regular data updates and continued data integration with relevant datasets. Future work includes semantic integration of AOP-DB disease-gene data, tissue-specific gene interaction networks, AOP functional single nucleotide polymorphism (SNP) and population SNP frequency information and chemical-specific datasets.

## Data Availability

The original contributions presented in the study are included in the article/Supplementary Material and are also made available at: https://github.com/BiGCAT-UM/AOP-DB-RDF. Further inquiries can be directed to the corresponding author.

## Abbreviations

AOP, Adverse Outcome Pathway; AOP-DB, Adverse Outcome Pathway Database; AOP-KB, Adverse Outcome Pathway Knowledgebase; AOP-Wiki, The Collaborative Adverse Outcome Pathway Wiki; FAIR, Findable, Accessible, Interoperable, Reusable; ID, Identifier; IRI, Internationalized Resource Identifier (IRI); KE, Key Event; MIE, Molecular Initiating Event; NCBI; National Center for Biotechnology Information; NRC, National Research Council; NAMs, New Approach Methodologies, OECD, Organisation for Economic Co-operation and Development; RDF, Research Description Framework; SPARQL, SPARQL Protocol and RDF Query Language; SQL, Standard Query Language; ToxCas, tEPA’s Toxicity ForecasterUS EPA, United States Environmental Protection Agency; W3C, World Wide Web Consortium.
